# Hand-made mesh traction band to improve lateral view in colorectal endoscopic submucosal dissection

**DOI:** 10.1055/a-2422-5958

**Published:** 2024-10-14

**Authors:** Tomoki Shimizu, Yusuke Takai, Eiji Sakai

**Affiliations:** 1 73906 Department of Gastroenterology, Yokohama Sakae Kyosai Hospital, Yokohama, Japan

In endoscopic submucosal dissection of large colorectal lesions, use of single traction results in a poor lateral view owing to the weight of the lesion. Conventional traction devices are not elastic, and use of additional traction clips induces excessive traction force and specimen damage, limiting the size of the lesions that can be accommodated 1 2 3 . Moreover, using multiple traction devices increases costs, complicates device removal procedures, and is time consuming 4 5 . No single device that allows multiple lateral tractions in any situation has been established. Therefore, we developed a novel device: the mesh traction band (MTB).

The MTB device was produced by cutting commercially available polyethylene nets. Polyethylene is a safe material with excellent chemical resistance and insulating properties, and is used in food packaging. It was cut into rectangular sections of 15 × 20 mm (five rhombi each for length and width) and inserted through the endoscope channel with thin forceps grasping the short side of the device. Thereafter, the midpoints of the long sides were clipped to the resected specimen flap and the anal side of the opposite intestinal wall, respectively, for traction ( Fig. 1 ). When necessary, lateral traction was added by clipping the rhombi at the left and right corners of the MTB to the specimen in the 4 and 8 o’ clock directions. The traction clips were pulled toward the center of the lumen, improving the view of the submucosa in all directions.

**Fig. 1:**
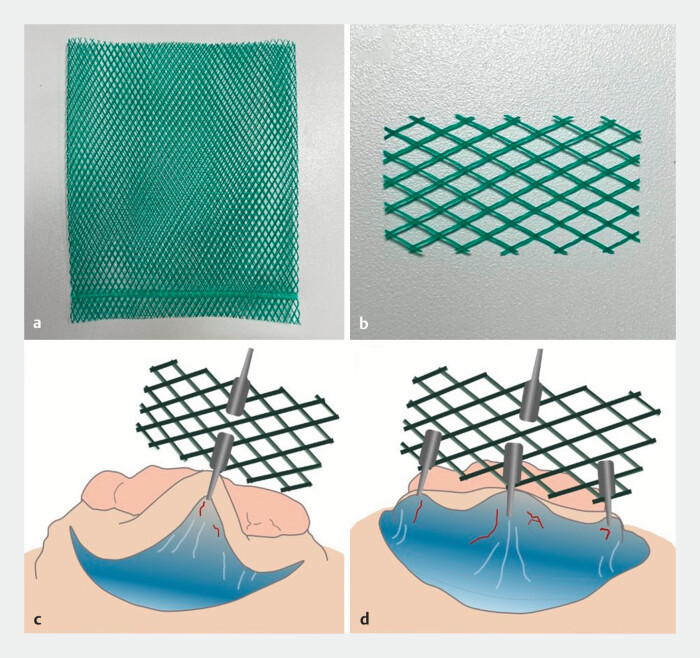
Use of the mesh traction band (MTB) for traction during resection. **a, b** A polyethylene net was cut into rectangular sections of five rhombi across both length and width. **c** The MTB was inserted into the lumen through the endoscope channel with forceps, and clipped at the midpoints of the long sides to the specimen and intestinal wall for traction. **d** Lateral traction was available by clipping the rhombi at the corners of the MTB to the specimen.

The advantage of the MTB is that it is applicable regardless of the size or location of the lesion, as the elasticity of the mesh structure absorbs excessive traction force. Video 1 shows a case in which the weight of the lesion interfered with the procedure. Additional lateral traction assisted in safe dissection.

Additional lateral traction with a mesh traction band in a case in which the weight of the lesion interfered with dissection.Video 1

The MTB is a novel device that facilitates additional lateral traction and reduces procedure difficulty in any situation ( Fig. 2 ).

**Fig. 2:**
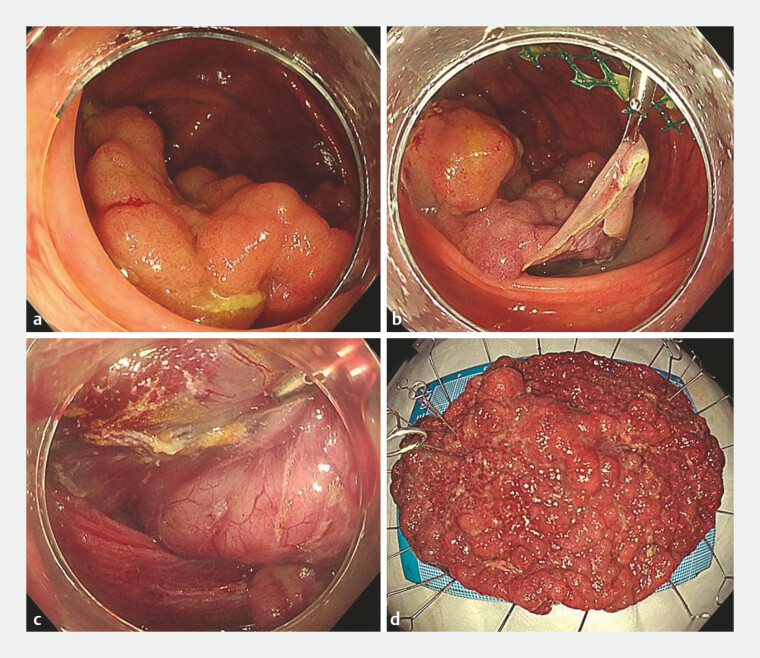
Endoscopy and specimen images. **a** A large laterally spreading tumor in the cecum, over 100 mm in size and involving the ileocecal valve. **b** With a single point traction alone, it was difficult to reach the submucosal layer on the ileocecal valve side owing to the weight of the tumor. **c** Additional traction on the left side improved the submucosal view and allowed dissection while checking the stereo architecture of the muscular layer. **d** The tumor was completely resected without damage to the muscle layer and specimen.

Endoscopy_UCTN_Code_TTT_1AQ_2AD_3AD

## Notes

Endoscopy E-Videos https://eref.thieme.de/e-videos
*E-Videos* is an open access online section of the journal *Endoscopy* , reporting on interesting cases and new techniques in gastroenterological endoscopy. All papers include a high-quality video and are published with a Creative Commons CC-BY license. Endoscopy E-Videos qualify for HINARI discounts and waivers and eligibility is automatically checked during the submission process. We grant 100% waivers to articles whose corresponding authors are based in Group A countries and 50% waivers to those who are based in Group B countries as classified by Research4Life (see: https://www.research4life.org/access/eligibility/ ). This section has its own submission website at https://mc.manuscriptcentral.com/e-videos .
